# Amplification of Vitamin D_2_ in the White Button Mushroom (*Agaricus bisporus*) by UV-B Irradiation and Jet-Milling for Its Potential Use as a Functional Ingredient

**DOI:** 10.3390/foods9111713

**Published:** 2020-11-22

**Authors:** Tae-Yeong Heo, Ye-Na Kim, Il Bum Park, Dong-Un Lee

**Affiliations:** 1 Department of Food Science and Technology, Chung-Ang University, Anseong 17546, Korea; xodud1485@naver.com (T.-Y.H.); kyn6874@naver.com (Y.-N.K.); 2 Yuhan Care, Ltd., Giheung-gu, Yongin 17084, Korea; joshua.park@yuhancare.com

**Keywords:** vitamin D_2_, UV-B irradiation, jet mill, superfine powder, white button mushroom

## Abstract

The objective of this study was to amplify vitamin D_2_ in white button mushrooms using ultraviolet (UV-B) irradiation and to prepare a vitamin D_2_-fortified superfine mushroom powder through jet milling. Mushrooms irradiated with UV-B for 30 min had a vitamin D_2_ concentration of 8.19 μg/g, an amount about 400 times greater than that of the control (0.02 μg/g). The vitamin D_2_-fortified mushrooms were then freeze-dried and conventionally ground or jet-milled to obtain coarse (Dv_50_ = 231 μm), fine (Dv_50_ = 106.3 μm), and superfine (Dv_50_ = 7.1 μm) powders. The vitamin D_2_ content was retained during the preparation of the powders. The physical characteristics were evaluated by scanning electron microscopy and hydration properties. The superfine powder of vitamin D_2_-amplified mushrooms was suitable for use as a functional ingredient because its roughness was significantly reduced, and it had a neutral aroma and taste as determined by descriptive analysis.

## 1. Introduction

Ergocalciferol (vitamin D_2_) is a synthetic form of vitamin D that can be formed by ultraviolet (UV) irradiation from the plant steroid ergosterol [1]. Vitamin D is also known to prevent some diseases, such as osteomalacia and rickets [2]. Disorders caused by vitamin D deficiency are frequently reported worldwide [3]. Mushrooms contain large quantities of ergosterol, which is converted to vitamin D_2_ when exposed to sunlight or ultraviolet light. The conversion of vitamin D was found to be dependent on the applied wavelength. The conversion was most effective under ultraviolet-B (UV-B) than ultraviolet-C (UV-C) or ultraviolet-A(UV-A) [4,5,6]. Vitamin D is commonly contained in animal-based foods, but mushrooms contain a high concentration of ergosterol, which can be converted to vitamin D [7]. Currently, the most cultivated and consumed mushroom is button mushrooms (*Agaricus bisporus*), which is very popular in both the East and the West [4]. The processing of UV-B-irradiated white button mushrooms is challenging because discoloration occurs during UV-B irradiation, and mushrooms decay immediately due to their high water content. An additional process is needed to extend the shelf life, and drying is frequently used to prolong the shelf life of mushrooms [8]. Dried mushrooms can be further processed into powder form, which can be added to various foods as a functional ingredient with unique flavors [9]. However, the unique flavor and fibrous texture of mushrooms limit the use of mushrooms as food ingredients because many people dislike the fibrous texture and flavor of mushrooms.

Particle size determines the organoleptic properties of a powder. Roughness is directly related to the particle size of the raw material, and the overall acceptance of a fiber-enriched layer cake decreases with an increase in fiber particle size [10]. The small powder size has a corresponding large surface area, and thus water absorption rate and solubility are high [11]. Particle sizes of the raw materials also affect the hydration properties as well as the extractability of food components [12,13]. Previous studies have shown that superfine mushroom powder has remarkable fluidity, water holding capacity and water solubility and is suitable for making instant foods [13,14]. Jet milling is a novel size reduction process, which creates micro and nanoscale particles with minimal thermal impact on food materials [15]. Jet milling is classified as impact milling. Particles are accelerated in a high-velocity air or gas stream, and size reduction is the result of inter-particle collisions or impacts against a solid surface. The final particle size produced by this method is very much dependent on the material being processed, with processing possible even at the 1000 nm scale [16,17].

The objectives of this study were to optimize the formation of vitamin D_2_ in white button mushroom through UV-B irradiation and to determine the effect of the superfine grinding process on the vitamin D_2_ concentration and physical properties of mushroom powders.

## 2. Materials and Methods

### 2.1. Materials

Freshly harvested white button mushroom (*Agaricus bisporus*) samples were obtained from a local mushroom farm (Nonsan, Korea) and stored at 4 °C before use. The samples were used in experiments within three days of storage. 

### 2.2. UV-B Irradiation of Mushrooms

The UV-B (ultraviolet-B) irradiation of mushrooms was performed using a UV-B irradiator (Ilsinnafyu, Seongnam, Korea) at room temperature. The irradiator was equipped with four Phillips UV-B lamps (TL40W/12; Phillips, Amsterdam, The Netherlands); the irradiation height was 7 cm. The UV-B output was about 12.5 W/m^2^. The mushrooms were sliced to an average thickness of 4 mm, and the sliced mushrooms have a moisture content of about 88%. The sliced mushrooms were placed on shelves and exposed to UV-B radiation for 10, 20, 30, 60 and 90 min. Five hundred grams of sliced mushrooms were used for each treatment. The irradiated samples were freeze-dried (LP 20; Ilshinbiobase, Dongducheon, Korea) and stored airtight in aluminum foil bags to protect against additional UV exposure.

### 2.3. Vitamin D_2_ and Ergosterol Analysis by High-Performance Liquid Chromatography (HPLC)

The vitamin D_2_ and ergosterol concentrations were determined using a method developed in a previous study, with some modifications [18]. A mushroom sample (1 g) was weighed into a 250 mL flask and then mixed with 1 g of *L*-ascorbic acid (Duksan Pure Chemicals Co., Ansan, Korea), 25 mL of 55% potassium hydroxide (Duksan Pure Chemicals Co.), 50 mL of 99% ethanol (Burdick & Jackson, Muskegon, MI, USA). The mixture was thoroughly shaken. The saponification was conducted for 30 min under reflux at 85 °C. The mixture was cooled immediately and transferred into a separatory funnel. The extraction was conducted with 30 mL of *n*-hexane (Daejung Chemicals & Metals, Siheung, Korea) and 10 mL of de-ionized water, and this procedure was repeated twice. The pooled organic layer was washed three times, with 25 mL of de-ionized water until it was neutralized. The organic layer was transferred into a round flask, rotary-evaporated to dryness at 50 °C, and immediately re-dissolved in 2 mL of a solution of isopropyl alcohol, methanol and acetonitrile (2:1:3, *v*/*v*). The samples were passed through a filter (PTFE, 13 mm; Whatman International, Ltd., Maidstone, UK), with a pore size of 0.45 μm. Table 1 shows the HPLC system conditions. A 20 μL sample volume was injected into an HPLC system (LC-4000 series; Jasco, Tokyo, Japan) and eluted through a reverse phase C18 column (Polaris 4.6 × 250 mm; Agilent, Santa Clara, CA, USA). The mobile phase was methanol/acetonitrile (75:25, *v*/*v*) at a flow rate of 1 mL/min. The detection wavelength of the UV-vis detector (UV-4075; Jasco, Tokyo, Japan) was at 245 nm. The content of vitamin D_2_ and ergosterol were calculated on the basis of the calibration curve of authentic vitamin D_2_ standard (Merck, Darmstadt, Germany) and ergosterol standard (Merck, Darmstadt, Germany).

### 2.4. Preparation of Coarse, Fine and Superfine Powders from Vitamin D_2_-Amplified Mushrooms

Mushrooms were UV-B irradiated for the optimal time of 30 min (determined as described in Section 3.2) to convert ergosterol into vitamin D_2_. The irradiated samples were freeze-dried (LP 20; Ilshinbiobase, Dongducheon, Korea) and thoroughly blended with a laboratory mixer. The resulting powder was serially sieved with 150 and 63 μm testing sieves (Nonaka Rikaki, Chiyoda, Japan) using a sieve shaker (EML200; Haver and Boecker, Oelde, Germany). The retained powders over the 150 μm sieve were classified as the coarse mushroom powder with vitamin D_2_. The 150 μm sieve passed powders, but retained over the 63 μm sieve were classified as the fine mushroom powder with vitamin D_2_. The blended mushroom powder was further milled by a fluidized-bed jet mill (CGS 10; NETZSCH, Selb, Germany). The milling pressure was 7 bars, and the classifier was set to 12,000 rpm. The jet-milled powder was collected as the superfine mushroom powder with vitamin D_2_. All powder samples were airtight packaged and stored at −20 °C before use.

### 2.5. Particle Size Distribution

The particle size of the mushroom powder was measured by a particle size analyzer (Mastersizer 3000; Malvern Instrument Limited, Worcestershire, UK), using the dry method, with a particle size absorption index of 0.1, particle refractive index of 1.53 and dispersant refractive index of 1.0.

### 2.6. Scanning Electron Microscopy (SEM)

The microstructure of the mushroom powder was examined by a scanning electron microscope (S-3400 N; Hitachi, Tokyo, Japan). Samples were affixed to carbon tape and coated with platinum-lead (Pt-Pb) before observation. The microstructure of the mushroom powder was observed under magnifications of 300×, 500× and 1000×.

### 2.7. Hydration Properties

The water holding capacity of the powder was measured according to a previously reported method [14], with some modifications. The mushroom powder $(M_{1}$, 0.5 g) was poured into a 50 mL centrifuge tube (M). Then, 20 mL of distilled water was added. The mixture was incubated at 60 °C for 30 min. The mixture was cooled in ice for 30 min. The cooled mixtures were then centrifuged at 10,000 rpm for 20 min. After the supernatant was removed, the weight of the residue powder and centrifuge tube ($M_{2})$ was recorded. Water holding capacity (g/g) was expressed by the following Equation (1):

Water holding capacity (g/g) = (*M*_1_ (g)/(*M*_2_ (g) − *M* (g)). (1)

The swelling capacity of the powder was measured as per a previous report with slight modification [14]. The mushroom powder (1 g) was transferred into a graduated falcon tube, and the bed volume was measured. Then 10 mL of distilled water was added and shaken. The created dispersion by shaken was incubated at 25 °C for 24 h. The final volume of the wetted powder was recorded. Swelling capacity (mL/g) was expressed by the following Equation (2):

Swelling capacity (mL/g) = (Final volume (mL) − Initial volume (mL))/Sample (g).(2)

The water solubility index was determined according to a previously reported method [14], with some modifications. The mushroom powder ($W_{1}$, 0.5 g) was dispersed in 25 mL of distilled water and poured into a 50 mL falcon tube. The sample tubes were incubated in a water bath at 80 °C for 30 min and then centrifuged at 10,000 rpm for 20 min. The supernatant liquid was collected in a preweighed aluminum dish ($W_{2}$) and dried at 105 °C for 24 h. The weight of the dried residue ($W_{3}$) was measured. The water solubility index (WSI) was expressed by the following Equation (3):

Water solubility index (%) = (*W*_3_ (g) − *W*_2_ (g))/(*W*_1_ (g) × 100). (3)

### 2.8. Sensorial Evaluation Using a Descriptive Analysis

A descriptive analysis method was applied to evaluate the sensory properties of mushroom powder [19,20]. For the sensory evaluation, powder samples (5 g) were placed in white dishes. The dishes were labeled with three-digit random numbers. Twelve panelists were selected from the graduate students of Chung-Ang University. Descriptors and definitions used for the sensory evaluation of vitamin D_2_ fortified mushroom powder are shown in Table 2. Three sensory attributes (roughness, mushroom aroma and mushroom taste) were evaluated to determine the sensorial property of mushroom powder.

### 2.9. Statistical Analysis of Data

All experiments were performed in triplicate. The results were expressed as the mean ± standard deviation. An analysis of variance (ANOVA) and Duncan’s multiple range comparison tests were performed using SPSS Statistics 19.0 software (IBM, New York, NY, USA), with *p*-values < 0.05 considered significant.

## 3. Results and Discussion

### 3.1. Appearance of UV-B-Irradiated Mushrooms

**Figure 1:**
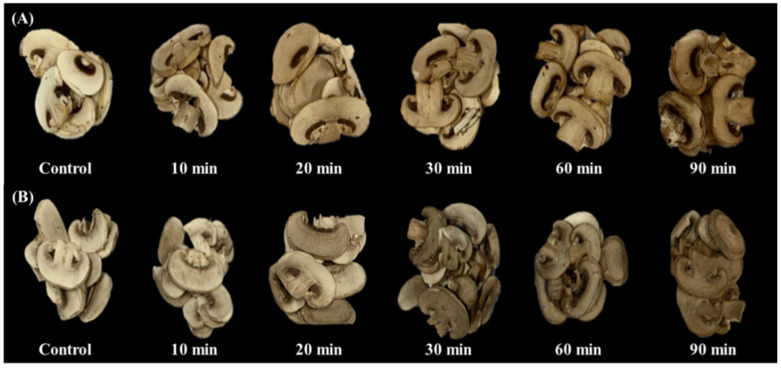
Sliced white button mushrooms (*Agaricus bisporus*) treated with UV-B for various times (**A**); the appearance of the control and UV-B-treated mushrooms after freeze-drying (**B**).

Figure 1 shows the appearance of UV-B-irradiated mushroom samples. Figure 1A shows the UV-B-irradiated mushrooms after various irradiation times, and Figure 1B shows the UV-B-irradiated mushroom samples after freeze-drying. There was a visible change in the appearance of the surface of the mushroom slices; the surface color changed from white to brown. A previous study indicated that UV-C irradiation also led to mushroom discoloration [21]. Long-term UV-B irradiation may result in degraded mushroom quality due to surface discoloration. The surface discoloration of mushrooms affects the acceptability of the product. The irradiated samples were freeze-dried, and the surface discoloration of the UV-B-treated mushrooms remained after freeze-drying (Figure 1B).

### 3.2. Effect of UV-B Irradiation on Vitamin D_2_ and Ergosterol Concentrations of Mushrooms

**Figure 2:**
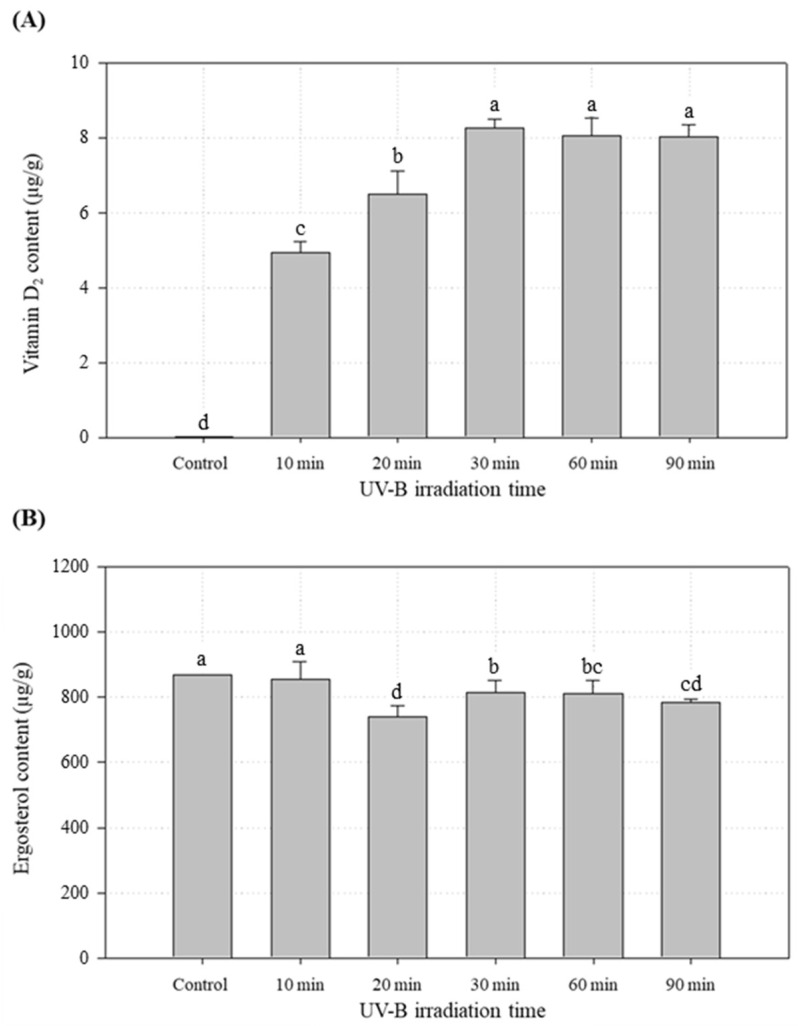
Effect of UV-B irradiation on the vitamin D_2_ (**A**) and ergosterol (**B**) concentrations in white button mushrooms. All values are expressed as the mean ± standard deviation of triplicate analyses. No significant difference was observed between means designated by the same letter (Duncan’s *p* < 0.05).

The effect of UV-B irradiation on the vitamin D_2_ and ergosterol concentrations of white button mushrooms is shown in Figure 2. The vitamin D_2_ concentration in the untreated control was 0.02 μg/g (Figure 2A). After exposure to UV-B for 10, 20 and 30 min, the vitamin D_2_ concentration increased to 4.93, 6.50 and 8.19 μg/g, respectively. These observations indicated that the conversion of ergosterol into vitamin D_2_ was dependent on the UV-B irradiation time in the early time points. However, UV-B irradiation times longer than 30 min did not further increase vitamin D_2_ concentration. After 30, 60 and 90 min of UV-B irradiation, the vitamin D_2_ concentrations were statistically identical (*p* < 0.05). Overall, the vitamin D_2_ concentration of mushrooms irradiated with UV-B for 30 min was about 400 times greater than that of the control and remained constant following further UV-B treatment. Therefore, the optimal UV-B irradiation time for the white button mushrooms was determined to be 30 min.

It has been suggested that only a small fraction of ergosterol is converted to vitamin D_2_ by UV irradiation [18]. The conversion of ergosterol to vitamin D_2_ in the early stage of the process is a typical zero-order reaction where the concentration of the reacting compound, i.e., ergosterol, is so large that the overall reaction rate appears to be independent of its concentration [22]. Our results support this explanation because there was little difference in the ergosterol concentration after UV-B irradiation (Figure 2B). The amount of ergosterol within the mushrooms was estimated to be a hundred-fold higher than that of vitamin D_2_.

### 3.3. Size Distributions of Coarse, Fine and Superfine Mushroom Powders

**Figure 3:**
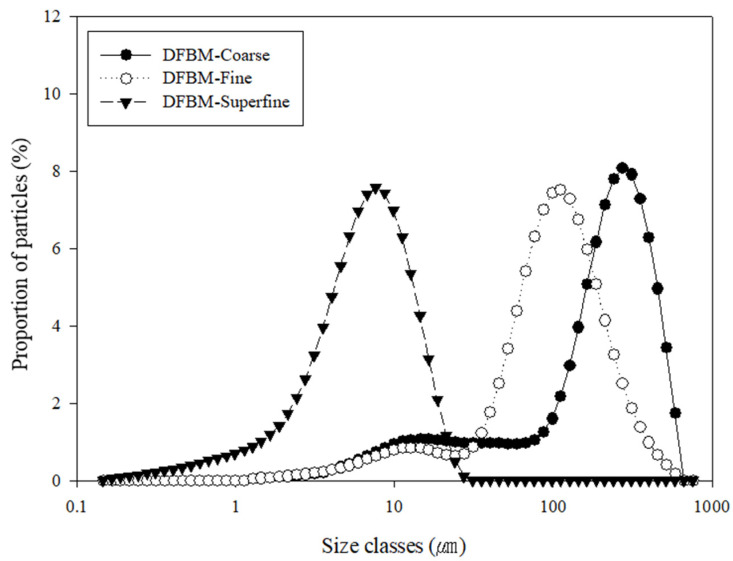
Particle size distribution of coarse, fine and superfine mushroom powders. The white button mushrooms were UV-B treated for 30 min and freeze-dried before pulverization.

The UV-B-treated mushrooms were freeze-dried and pulverized into coarse, fine and superfine powders. The particle size distributions of the mushroom powders are shown in Table 3. The results are presented as the equivalent diameter at a cumulative volume of 10% (Dv_10_), 50% (Dv_50_) and 90% (Dv_90_). The Dv_50_ values of the coarse, fine and superfine powders were 231.0 ± 1.7, 106.3 ± 2.1 and 7.1 ± 0.3 μm, respectively. The specific surface area of the mushroom powders increased as the particle size decreased. The results indicated that jet-milling effectively pulverized the mushroom into a superfine scale of <10 mm and confirmed the homogeneous size distribution of superfine powder (Figure 3).

The reduced particle size of the superfine mushroom powder is beneficial for its use as a food ingredient because it minimizes the rough mouthfeel and fibrous texture. The application of jet milling successively can reduce the particle size of fibrous material below 10 μm, which has been reported for soybean flour [11], the insoluble fiber-rich fraction of carrot [23] and mushroom stem and cap powders [14].

### 3.4. Microstructure of Coarse, Fine and Superfine Mushroom Powders

**Figure 4:**
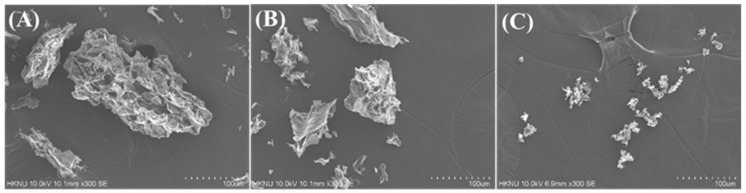
Scanning electron micrographs of (**A**) coarse, (**B**) fine and (**C**) superfine mushroom powders at 300× magnification. The white button mushrooms were UV-B treated for 30 min and freeze-dried before pulverization.

The microstructure of mushroom powders was observed using SEM (Figure 4). The coarse and fine mushroom powders that were prepared by a conventional mixer followed by sifting (Figure 4A,B) had a flatter, flakier and more irregular shape because the mushrooms were mainly composed of fibers and carbohydrates [13]. On the other hand, the particle size of jet-milled, superfine mushroom powder (Figure 4C) was significantly smaller than that of the other samples. The superfine mushroom powder had a less flaky shape and was more homogenous in size. Homogeneity is a desirable property for food ingredients, and the jet milling process imparts a physical functionality to mushroom powder.

### 3.5. Vitamin D_2_ Concentrations of Coarse, Fine and Superfine Mushroom Powders

**Figure 5:**
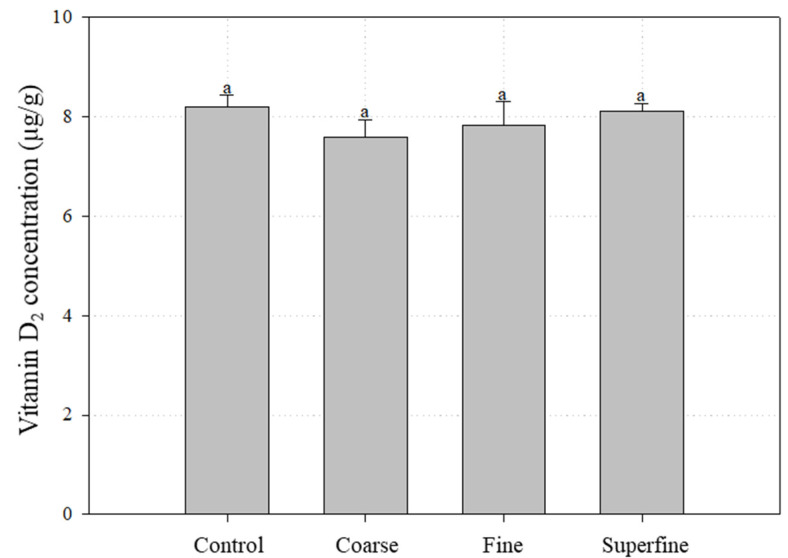
Effect of particle size reduction on the vitamin D_2_ concentration of mushroom powders. The control mushrooms were UV-B irradiated (30 min) and freeze-dried. Coarse fine and superfine mushroom powders were prepared using the control mushrooms. All values were expressed as the mean ± standard deviation of triplicate analysis. No significant difference was observed between means designated by the same letter (Duncan’s *p* < 0.05).

The vitamin D_2_ concentrations of the coarse, fine and superfine mushroom powders were 7.6 ± 0.3, 7.8 ± 0.5 and 8.1 ± 0.1 μg/g, respectively (Figure 5). The vitamin D_2_ concentration of unpulverized mushrooms was 8.2 ± 0.3 μg/g. There was no statistical difference among the samples. The results showed that there was no loss of vitamin D_2_ due to the grinding of the mushrooms, including the jet milling process.

According to a previous study, vitamin D_2_ from irradiated white button mushrooms is strongly absorbed and metabolized, as demonstrated by the 25-hydroxyvitamin D serum response to rats fed with the irradiated mushrooms [21]. It has been predicted that the amplified vitamin D_2_ within mushroom powders could be efficiently absorbed and metabolized.

### 3.6. Hydration Properties of the Coarse, Fine and Superfine Mushroom Powders

Table 4 shows the hydration properties of the mushroom powders with different particle sizes. The water holding capacity decreased significantly with decreasing particle size (*p* < 0.05). The effect of reduced particle size on the water holding capacity of a powder remains controversial. The water holding capacity in superfine silver carp bone powder was found to be higher than in coarser powders [24], whereas the superfine grinding of wheat bran resulted in a reduced water holding capacity [25]. Our previous studies also produced contradictory results. The jet milling of defatted soybean powder resulted in a significant increase in water holding capacity [11], whereas the same procedure resulted in a significant decrease in the water holding capacity of *Hericium erinaceum* [13]. It is likely that jet milling induces an increase in water holding capacity by increasing the surface area; however, at the same time, it also induces a decrease in the water holding capacity by breaking down the complexity of the microstructure. Our current results indicate that the breakdown of complexity is a more important factor than the increase in the surface area following the jet milling of mushroom powder, which results in a reduced water holding capacity.

The swelling capacity indicates how well the sample expands by absorbing water. The results indicate that coarse mushroom powders absorb more water and swell more than fine mushroom powders.

The water solubility index represents how well a sample is dissolved in water. The water solubility index of mushroom powder increased with decreasing particle size, with superfine mushroom powder having the largest water solubility index value of 54.19 ± 1.08%. According to a previous study, the water solubility index increases with decreasing particle size, which is due to the increased surface area and improved solubilization of protein [24]. This means that superfine mushroom powder is more soluble in water than coarser powders and would therefore be easier to use as a food ingredient in processed food.

### 3.7. Sensory Evaluation of the Coarse, Fine and Superfine Mushroom Powders

The sensory properties of the mushroom powders were analyzed by a descriptive analysis method (Table 5). Three sensory attributes (roughness, mushroom aroma and mushroom taste) were assessed alongside previously identified standards, as shown in Table 2.

Roughness was defined as the mouthfeel of the particles. As expected, roughness decreased significantly as the particle size of mushroom powder decreased (*p* < 0.05).

The aroma and taste of mushrooms also decreased significantly as the particle size of the mushroom powder decreased. The reduction in mushroom aroma and taste in superfine mushroom powder was unexpected because the increased surface area of superfine powder was expected to possibly accelerate the leaching of aroma and taste compounds. One possible explanation for this unexpected observation is that the compounds responsible for the mushroom aroma and taste were partly removed during the size reduction and jet milling processes. The acceptability of a mushroom’s aroma and the taste is strongly dependent on personal preference. These results show that superfine mushroom powder can be a good natural source of vitamin D_2_ for consumers who dislike mushrooms because it exhibits a weaker mushroom aroma and taste than other powders. The results indicated that the superfine mushroom powder with vitamin D_2_ could be applied to processed foods such as beverages, soups or and confectionaries, where the sensorial properties determine the acceptability of products.

## 4. Conclusions

The nutritional and physical properties of the white button mushroom were tailored through the consecutive physical application of UV-B irradiation and jet milling. Exposure to UV-B effectively increased the vitamin D_2_ concentration of mushrooms by up to 400 times after 30 min of irradiation. Vitamin D_2_-fortified mushrooms were then sized into coarse (Dv_50_ = 231.0 μm), fine (Dv_50_ = 106.3 μm) and superfine (Dv_50_ = 7.1 μm) powders. There were no significant changes in the vitamin D_2_ concentration following the grinding process. Through a descriptive analysis, the superfine powder was confirmed to have reduced roughness and weaker mushroom aroma and taste compared to the other samples. These results indicate that the UV-B-treated superfine mushroom powder can be a good natural source of vitamin D_2_ for consumers who dislike mushrooms because it exhibits a weaker mushroom aroma and taste than other powders.

## Figures and Tables

**Table 1:** Characteristics of the high-performance liquid chromatography (HPLC) system used.

Liquid Chromatography	
Model	LC-4000 series, Jasco, Japan
Column	Reverse phase C 18 column (Polaris 4.6 × 250 mm)
Column temperature	25 °C
Flow rate	1 mL/min
Detection wavelength	245 nm
Injection volume	20 μL
Mobile phase	Methanol:acetonitrile = 75:25 (*v*/*v*)

**Table 2:** Descriptors used for the sensory evaluation of vitamin D_2_ fortified mushroom powder by descriptive analysis.

Descriptor	Definition	Strength/Standard Material	
		1	9
Roughness	Mouthfeel of the particles	Small	Large
		Plain yogurt	Perilla seed powder
Mushroom Aroma	Aroma of cooked white button mushroom	Weak	Strong
		Water	White button mushroom (cooked)
Mushroom Taste	Taste of cooked white button mushroom	Weak	Strong
		Water	White button mushroom (cooked)

**Table 3:** Particle size and specific surface area of coarse, fine and superfine powders produced from white button mushrooms exposed to a 30-min UV-B treatment.

Sample	Dv_10_ (μm)	Dv_50_ (μm)	Dv_90_ (μm)	Specific Surface Area (m^2^/kg)
Coarse mushroom powder	20.3 ± 1.8 ^b^	231.0 ± 1.7 ^a^	456.3 ± 1.5 ^a^	387.1 ± 22.4 ^b^
Fine mushroom powder	23.1 ± 0.9 ^a^	106.3 ± 2.1 ^b^	245.0 ± 8.2 ^b^	460.1 ± 14.8 ^b^
Superfine mushroom powder	2.1 ± 0.2 ^c^	7.1 ± 0.3 ^c^	15.1 ± 0.4 ^c^	5345.7 ± 251.3 ^a^

All values are expressed as the mean ± standard deviation of triplicate analyses. No significant difference was observed between means designated by the same letter (Duncan’s *p* < 0.05).

**Table 4:** Hydration properties of coarse, fine and superfine mushroom powders produced from white button mushrooms exposed to a 30 min UV-B treatment.

Sample	Water Holding Capacity (g)	Swelling Capacity (mL/g)	Water Solubility Index (%)
Coarse mushroom powder	5.25 ± 0.20 ^a^	10.92 ± 0.50 ^a^	46.69 ± 2.33 ^b^
Fine mushroom powder	4.64 ± 0.11 ^b^	8.20 ± 0.12 ^b^	49.35 ± 2.04 ^b^
Superfine mushroom powder	3.13 ± 0.08 ^c^	4.56 ± 0.40 ^c^	54.19 ± 1.08 ^a^

All values are expressed as the mean ± standard deviation of triplicate analyses. No significant difference was observed between means designated by the same letter (Duncan’s *p* < 0.05).

**Table 5:** Sensory properties of coarse, fine and superfine mushroom powders produced from white button mushrooms exposed to a 30 min UV-B treatment.

Sample	Roughness	Mushroom Aroma	Mushroom Taste
Coarse mushroom powder	7.11 ± 1.11 ^a^	5.68 ± 1.06 ^a^	5.84 ± 1.17 ^a^
Fine mushroom powder	4.05 ± 1.03 ^b^	4.95 ± 1.22 ^b^	4.63 ± 0.90 ^a^
Superfine mushroom powder	2.53 ± 0.70 ^c^	3.00 ± 1.00 ^c^	3.21 ± 1.03 ^b^

All values are expressed as the mean ± standard deviation of triplicate analyses. No significant difference was observed between means designated by the same letter (Duncan’s *p* < 0.05).
